# Safety and Efficacy of Chest Physiotherapy in Patients With COVID-19: A Critical Review

**DOI:** 10.3389/fmed.2020.00454

**Published:** 2020-07-21

**Authors:** Auwal Abdullahi

**Affiliations:** ^1^Department of Physiotherapy, Bayero University Kano, Kano, Nigeria; ^2^Department of Physiotherapy and Rehabilitation Sciences, University of Antwerp, Antwerp, Belgium

**Keywords:** physiotherapy, pneumonia, COVID-19, mortality, ventilator, critical care

## Abstract

The present global pandemic of COVID-19 has brought the whole world to a standstill, causing morbidity, death, and changes in personal roles. The more common causes of morbidity and death in these patients include pneumonia and respiratory failure, which cause the patients to require artificial ventilation and other techniques that can improve respiratory function. One of these techniques is chest physiotherapy, and this has been shown to improve gas exchange, reverse pathological progression, and reduce or avoid the need for artificial ventilation when it is provided very early in other respiratory conditions. For patients with COVID-19, there is limited evidence on its effect, especially in the acute stage and in patients on ventilators. In contrast, in patients after discharge, chest physiotherapy in the form of respiratory muscle training, cough exercise, diaphragmatic training, stretching exercise, and home exercise have resulted in improved FEV1 (L), FVC (L), FEV1/FVC%, diffusing lung capacity for carbon monoxide (DLCO%), endurance, and quality of life, and a reduction in anxiety and depression symptoms. However, there are still controversies on whether chest physiotherapy can disperse aerosols and accelerate the rate of spread of the infection, especially since COVID-19 is highly contagious. While some authors believe it is possible, others believe the aerosol generated by chest physiotherapy is not within respirable range. Therefore, measures such as the use of surgical masks, tele-rehabilitation, and self-management tools can be used to limit cross-infection.

## Introduction

Coronavirus 2019 (COVID-19), more recently known as SARS-COV-2, is a coronavirus that belongs to the β-corona cluster that is spread to a large extent via droplets (1). When one contracts the infection, the virus gets into the lungs and is received by angiotensin-converting enzyme 2 (ACE2), which is expressed in normal humans in types I and II alveolar cells (2). When the virus binds with ACE2, it damages the alveolar cells (1). The alveolar cells function under normal circumstances to synthesize and secrete surfactant, carry out xenobiotic metabolism, help with transepithelial movement of water, and regenerate alveolar epithelium following lung injury (3). These aforementioned functions help with normal lung functions. Therefore, damage to the alveolar cells may result in respiratory problems, other systemic manifestations, and eventually death (1, 4). Consequently, clinical manifestations of COVID-19 disease include fever, cough, myalgia or fatigue, pneuomia, and complicated dyspnea (4).

In addition, when the respiratory symptoms are severe, they may progress to respiratory failure (acute respiratory distress syndrome), which could lead to death unless it is managed promptly using invasive ventilation (4–6). However, for those with mild to moderate symptoms, non-invasive techniques such as chest physiotherapy can be used (6). The aim of this article is to critically review the nature of respiratory problems in patients with COVID-19 and the safety and efficacy of the use of chest physiotherapy for these patients. Therefore, for the purpose of this review, PubMed and Google Scholar were searched using COVID-19, chest physiotherapy, and pulmonary rehabilitation as keywords.

## The Nature of Acute Respiratory Distress Syndrome in Patients With COVID-19

Acute respiratory distress syndrome (ARDS) in COVID-19 usually begins a bit later than in other ARDS. The onset is usually between 8 and 12 days after infection (7–9). It is usually characterized by a dry cough, which is said to be due to respiratory epithelial cells being affected more than the endothelial cells (10). Consequently, the patients usually present with mild symptoms of cough and dyspnea with no exudation that are inconsistent with laboratory and imaging findings including the presence of ground-glass opacities and basilar opacities and lymphocytopenia (8, 10–15). In contrast, some patients, especially those with comorbidities such as neuromuscular disorders and chronic obstructive pulmonary disorders, may at the same time or later develop exudative consolidation and mucous hyper-secretion along with difficulty in clearing the secretions (15). However, even in those presenting with mild symptoms, the dyspnea may progress quite rapidly and cause the patients to require ventilation (12, 16). Therefore, it is important to be on the alert and ready to use a ventilator for the patients if available. If ventilators are not available, other non-invasive techniques such chest physiotherapy can be used on a case-by-case basis (6, 17).

## Effects and Safety of Chest Physiotherapy in Patients With Other Respiratory Conditions and COVID-19

Chest physiotherapy has been used in many different respiratory conditions. It has been said to improve gas exchange, reverse pathological progression, and reduce or avoid the need for artificial ventilation when it is provided very early (18, 19). However, for patients with COVID-19, evidence is still lacking on its effects, especially during the acute stage, aside from some position papers or recommendations based on anecdotal evidence (15, 17, 20). This is because the features of respiratory problems in patients with COVID-19 significantly differ from those in other respiratory conditions. For instance, during the acute stage, patients with COVID-19 do not usually have exudation (10, 15). In addition, dyspnea in patients with COVID-19 may rapidly progress to acute respiratory failure (4, 5). Consequently, timely use of mechanical ventilation in such situations is strongly recommended (15, 17, 20).

COVID-19 is highly infectious and spreads rapidly, and there have been concerns about the use of chest physiotherapy in infectious diseases. This is because it has been argued that chest physiotherapy may cause aerosolization (21). This may increase the rate at which COVID-19 spreads. However, later findings in similar conditions disproved this view. According to Simonds and colleagues, an evaluation of droplet dispersion in Influenza pandemic and other airborne infections showed that chest physiotherapy significantly and predominantly produced droplets of >10 μm (22). Droplets of this size are not respirable, as only droplets within inspirable range (about 5 μm) can play a significant role in the transmission of infections (23). Similarly, a review of aerosol transmission of Influenza A virus cast doubt on whether it is even possible for droplets from chest physiotherapy to transmit infections (24). In addition, in SARS, a disease that shared similar pathophysiology with COVID-19 (7, 25), chest physiotherapy was later recommended (26). Furthermore, overall, the management of COVID-19 is as yet symptomatic, as scientists are still trying to understand its pathophysiology and the viral behavior (1, 27). Therefore, since the disease can kill within days to a month of onset, especially in the elderly and those with weak immunity (9, 28), we can make our patients sneeze or cough out sputum into disposable plastic bags during and after chest physiotherapy to prevent or reduce the chance of aerosolization. A similar measure was recommended previously (20).

Similarly, we can disinfect the surrounding environment, while at the same time, the therapists and other health workers wear protective gear to protect themselves from infection. In this way, we can save the lives of tens of thousands of people who might be infected with COVID-19. However, if surgical masks are available, the patients can wear them during the procedure to prevent the spread of the infection (17). Use of surgical masks and oxygen masks on the face of the patients has been found to deflect aerosols during chest compression in a simulation and cadaver model (29). In addition, other means such as the use self-management techniques such as the provision of self-management pamphlets and educational videos or consultations online, such as via Skype video communications, to help patients manage themselves 24 h a day can be explored (20). This will help reduce the chances of cross-infection.

For patients with COVID-19, the aim of chest physiotherapy is to alleviate dyspnea and relieve anxiety and depression in the short term (6, 20); in the long term, it is to improve physical functions, which will in turn improve quality of life and aid return to society (6, 20). Consequently, the chest physiotherapy interventions recommended and/or used for patients with COVID-19 include airway clearance techniques (active cycle of breathing technique, forced expiratory technique, percussion and vibrations, positive expiratory pressure (PEP) therapy (including bubble PEP), positioning and gravity-assisted postural drainage, intra- or extrapulmonary high-frequency oscillation devices, autogenic drainage), secretion clearance removal (huff and cough, suctioning, assisted or stimulated cough maneuvers, cough assist machine), and mobilization and exercise prescription, which may trigger a cough and/or sputum expectoration (15, 30). However, it has been recommended that rehabilitation should be provided on a case-by-case basis since patients differ in their clinical characteristics (15, 17, 20).

## Chest Physiotherapy During the Acute Period

During this stage, most patients have no exudation, and as such, chest physiotherapy may not be recommended (15). In addition, procedures such as diaphragmatic breathing, pursed-lip breathing, and bronchial hygiene/ lung re-expansion techniques are contraindicated during this stage (17). The priority here is the use of a mechanical ventilator, especially in those with severe symptoms (6, 17). For those with exudation and mild to moderate symptoms, it has been argued that chest physiotherapy can be used to relieve dyspnoea and depression and anxiety on a case-by-case basis (6, 15). However, to date, there are no studies reporting on the use of chest physiotherapy during the acute stage aside from a recommendation based on anecdotal evidence (17).

## Chest Physiotherapy During Mechanical Ventilation

Under mechanical ventilation, patients may lose spontaneous breathing (31). This can predispose the patients to developing lung collapse and ventilator-associated pneumonia. In such circumstances, chest physiotherapy can be used to reduce the length of stay in both a mechanical ventilator and ICU and prevent ventilator-associated pneumonia (32, 33). In addition, high-frequency chest wall oscillation for intubated patients resulted in increased dry sputum weight and PaO_2_ on day 3, decreased lung collapse on days 2 and 3, and culture positivity on day 3 (33). Similarly, in a patient who received 11 sessions of physical therapy consisting of upright body positioning, mobilization and exercise, and the active cycle of breathing exercise technique every 2 h for 12 h over his 48-h stay in the ICU (six sessions on day one and five sessions on day two), arterial oxygen level improved markedly, with radiographic resolution of infiltration (18). Therefore, since chest physiotherapy reverses pathological progression, prevents atelectasis, improves impaired gas exchange, and decreases culture positivity, which are also some of the pathological hallmarks of COVID-19, it can be utilized in patients with this disease.

Accordingly, the techniques recommended in patients who are on a ventilator include airway clearance techniques, lung maneuver recruitment, endotracheal suctioning, and change in posture (17, 20). The airway clearance techniques recommended include positioning, active cycle of breathing, manual and/or ventilator hyperinflation, percussion and vibration, positive expiratory pressure (PEP), and mechanical insuflation-ensufflation (15, 17). However, there are no details on how to perform these techniques aside from positioning therapy, and there have been no studies yet in patients with COVID-19 reporting on the efficacy of the techniques. See Table 1 for details of the positioning therapy technique. In addition, lung maneuver recruitment needs to be used with caution since it may have severe adverse effects (34). Furthermore, chest physiotherapy during this period is indicated or contraindicated based on the status of the respiratory, cardiovascular, and neurological functions of the patients. See Table 2 for details of the indications and the contraindications.

**Table 1:** Description of the chest physiotherapy (positioning therapy) used in patients with COVID-19 during mechanical ventilation [adopted from (17)].

**Procedure**	**Description**
Positioning therapy	Patients can be put in a prone position, 12–16 h per day, preferably within 72 h of endotracheal intubation. If this is effective, it should be repeated until PaO_2_/FiO_2_ ratio (P/F) ≥150 mmHg with PEEP ≤0.60 for at least 4 h in a supine position
Active cycle of breathing (airway clearance technique)	Nil
Manual and/ or ventilator hyperinflation (airway clearance technique)	Nil
Percussion and vibration (airway clearance technique)	Nil
Positive expiratory pressure (PEP) (airway clearance technique)	Nil
Mechanical insuflation-ensufflation (airway clearance technique)	Nil
Lung maneuver recruitment	Nil
Endotracheal suctioning	Nil

**Table 2:** Indications and contraindications of chest physiotherapy in patients in mechanical ventilations [adopted from (15, 17, 20)].

	**Respiratory function**	**Cardiovascular function**	**Nervous system function**	**Other**
Indication	Fraction of inspired oxygen (FiO2) ≤0.6, blood oxygen saturation (SpO2) ≥90%, respiratory rate ≤40 breaths/min, positive end expiratory pressure (PEEP) ≤10 cmH2O (1 cmH_2_O=0.098 kPa), absence of ventilator resistance, absence of unsafe hidden airway problems, difficulty breathing, severe or productive coughing episodes, and CT or lung ultrasound changes consistent with consolidation	Systolic blood pressure ≥90 mmHg or ≤180 mmHg, mean arterial pressure (MAP) ≥65 mmHg or ≤110 mmHg, heart rate ≥40 beats per minute (bpm) or ≤120 bpm, absence of new arrhythmia or myocardial ischemia, absence of shock with lactic acid level ≥4 mmol/L, absence of new unstable deep-vein thrombosis and pulmonary embolism, and absence of suspected aortic stenosis	Richmond Agitation-Sedation Scale (RASS) score: −2 to +2 and intracranial pressure <20 cmH_2_O	Absence of unstable limb and spinal fractures, absence of severe underlying hepatic/renal disease or new progressively worsening hepatic/renal impairment, absence of active hemorrhage, and temperature ≤38.5°C
Contraindication/ Discontinuation of Intevention	A low-level oxygen requirement (e.g., oxygen flow < 5 l/min for SpO_2_ > 90%) Blood oxygen saturation <90% or decrease of >4% from baseline, respiratory rate >40 breaths/min, ventilator resistance, and artificial airway dislodgement or migration	Systolic blood pressure <90 mmHg or >180 mmHg, MAP <65 mmHg or >110 mmHg or >20% change compared with baseline, heart rate <40 bpm or >120 bpm, and new arrhythmia and myocardial ischemia	Loss of consciousness and irritability	Discontinuation of any treatment or removal of monitoring tube connected to the patient, patient-perceived heart palpitations, exacerbation of dyspnea or shortness of breath, and intolerable fatigue, and falls in patient

*MAP, mean arterial pressure*.

## Post-Extubation and After Discharge

Post-extubation, many patients may develop respiratory failure again (35). This can be prevented using chest vibration and percussion (36). In patients with COVID-19, there seem to be no reports on the use of chest physiotherapy immediately post-extubation. However, following discharge, rehabilitation involving respiratory muscle training, cough exercise, diaphragmatic training, stretching exercise, and home exercise has been applied (30). These forms of training and exercise, when performed for two sessions per week for 6 weeks, resulted in improved FEV1 (L), FVC (L), FEV1/FVC%, diffusing lung capacity for carbon monoxide (DLCO%), endurance, and quality of life and a reduction in anxiety and depression symptoms. See Tables 3, 4 for the indications and contraindications of chest physiotherapy and descriptions of the techniques used during this stage, respectively.

**Table 3:** Indications and contraindications of chest physiotherapy in patients in after discharge [adopted from (20, 30)].

	**Respiratory function**	**Cardiovascular function**	**Nervous system function**	**Other**
Indication	A blood oxygen saturation of ≤95%, mini-mental state examination (MMSE) score > 21, no COPD or any other respiratory disease, and forced expiratory volume in 1s (FEV1) 70%	A heart rate of >100 beat per minute and a blood pressure of <90/60 mmHg or >140/90 mmHg	Nil	Other diseases that are not suitable for exercise
Contraindication/ Discontinuation of Intervention	Exacerbation of respiratory symptoms and fatigue that are not alleviated with rest, dyspnea, severe cough, moderate or severe heart disease, and ischemic or hemorrhagic stroke or neurodegenerative diseases	Chest tightness, chest pain, and heart palpitations	Dizziness, headache, blurred vision	Temperature fluctuation (>37.2°C), profuse sweating, and unstable gait

*COPD, chronic obstructive pulmonary disease*.

**Table 4:** Description of the chest physiotherapy used in patients with COVID-19 after discharge [adopted from (30)].

**Procedure**	**Description**
Respiratory training	Patients should use a hand-held resistance device for three sets, with 10 breaths in each set. Parameters should be set at 60% of the individual's maximal expiratory mouth pressure with a rest of 1 min between the three sets
Cough exercise	Three sets of active coughs should be adopted for cough exercises
Diaphragmatic training	Patients should perform 30 maximal voluntary diaphragmatic contractions in the supine position by placing a medium weight (1–3 kg) on the anterior abdominal wall to resist diaphragmatic descent
Stretching exercise	The respiratory muscles should be stretched under the guidance of a rehabilitation therapist. The patient should be placed in the supine or lateral decubitus position with the knees bent to correct the lumbar curve. Patients should be ordered to move their arms in flexion, horizontal extension, abduction, and external rotation
Home exercise	Patients should be instructed in pursed-lip breathing and coughing training. They should carry out 30 sets per day

## Conclusion

Chest physiotherapy may improve respiratory functions and quality of life in patients with COVID-19, especially after discharge. During the acute stage, evidence is still lacking on its usefulness, aside from some professional recommendations based on anecdotal evidence. However, it should be noted that chest physiotherapy is an individualized treatment based on the patient's particular presentations. Therefore, when patients present with symptoms that can benefit from chest physiotherapy, it may be given while the patients are closely observed for any adverse events. In addition, when administering chest physiotherapy for patients in the acute stage, measures such as the use of surgical masks, if available, should be taken to prevent cross-infection.

## Author Contributions

AA contributed solely to the conception, writing, and all sections of this article.

## Conflict of Interest

The author declares that the research was conducted in the absence of any commercial or financial relationships that could be construed as a potential conflict of interest.
